# Physical Activity, Rather Than Diet, Is Linked to Lower Insulin Resistance in PCOS Women—A Case-Control Study

**DOI:** 10.3390/nu15092111

**Published:** 2023-04-27

**Authors:** Justyna Jurczewska, Joanna Ostrowska, Magdalena Chełchowska, Mariusz Panczyk, Ewa Rudnicka, Marek Kucharski, Roman Smolarczyk, Dorota Szostak-Węgierek

**Affiliations:** 1 Department of Clinical Dietetics, Faculty of Health Sciences, Medical University of Warsaw, E Ciołka 27, 01-445 Warsaw, Poland; jjaniszewska@wum.edu.pl (J.J.); dorota.szostak-wegierek@wum.edu.pl (D.S.-W.); 2 Department of Screening Tests and Metabolic Diagnostics, Institute of Mother and Child, Kasprzaka 17a, 01-211 Warsaw, Poland; magdalena.chelchowska@imid.med.pl; 3 Department of Education and Research in Health Sciences, Faculty of Health Science, Medical University of Warsaw, 00-581 Warsaw, Poland; mariusz.panczyk@wum.edu.pl; 4 Department of Gynecological Endocrinology, Medical University of Warsaw, Karowa 2, 00-315 Warsaw, Poland; ewa.rudnicka@poczta.onet.pl (E.R.); marekkucharski@outlook.com (M.K.); rsmolarczyk@poczta.onet.pl (R.S.)

**Keywords:** polycystic ovary syndrome, insulin resistance, adipokines, HOMA-IR, HOMA-AD, L/A ratio, physical activity, diet

## Abstract

Insulin resistance (IR) is a prominent feature of polycystic ovary syndrome (PCOS). The importance of lifestyle interventions in the management of PCOS is strongly highlighted and it is suggested that diet and physical activity may significantly influence insulin sensitivity. Therefore, we evaluated the link between diet and physical activity and various indices of insulin resistance, including adipokines secreted by the adipose tissue in 56 PCOS and 33 healthy control women. The original food frequency questionnaire and Actigraph GT3X-BT were used to assess the adherence to the diet recommended in IR and the level of physical activity, respectively. We observed that higher levels of physical activity were associated with lower HOMA-IR and a greater chance of its normal value in PCOS group. No such relationship was observed for other IR indices and adipokines or for the diet. However, we noted a strong correlation between HOMA-IR (Homeostatic Model Assessment of Insulin Resistance) and HOMA-AD (Homeostatic Model Assessment-Adiponectin) in PCOS women. Additionally, when we used HOMA-AD we observed a higher prevalence of IR among PCOS women. Our study supports the beneficial role of physical activity in the management of insulin resistance in PCOS women. Moreover, our findings indicate that HOMA-AD may be a promising surrogate marker for insulin resistance assessment in women with PCOS.

## 1. Introduction

Polycystic ovary syndrome (PCOS) is one of the most common endocrine disorders affecting women of reproductive age. It is a complex condition commonly associated with anovulation, hyperandrogenemia (clinical or biochemical) and/or polycystic ovary morphology [1]. The prevalence of PCOS varies from 8 to 13% depending on the used diagnostic criteria [2]. 

Until recently, PCOS had only been considered as a cause of reproductive failure; however, it is now well-acknowledged that PCOS women are particularly susceptible to the development of many cardiometabolic disorders. Additionally, PCOS is strongly associated with obesity, which has an impact on the metabolic and reproductive complications in the course of this disease. As a result of hyperandrogenism, PCOS women are more likely to have abdominal obesity with increased content of visceral or subcutaneous fat compared to healthy women. Interestingly, the excessive accumulation of the visceral adipose tissue is also reported in PCOS women with normal body weight; thus, this parameter is not only related to BMI (body mass index). In addition, such fat distribution is strongly linked to hyperglycemia, compensatory hyperinsulinemia and insulin resistance [3,4]. Moreover, the adipose tissue is a very crucial and active endocrine organ which synthesizes and secretes a variety of adipokines influencing the regulation of metabolism related to the sensitivity of tissues to insulin [5]. The adipose tissue of women with PCOS shows many abnormalities in the secretion of adipokines which also play a significant role in the course of polycystic ovary syndrome. Some of these changes are characterized by increased serum concentrations of leptin and resistin and reduced expression of adiponectin. However, it is still unknown whether the anomalies are secondary to obesity, insulin resistance and hormonal imbalance or if they are inherent in the course of PCOS [6].

Insulin resistance (IR) is defined as reduced insulin sensitivity and refers to an increased amount of insulin needed to perform its metabolic actions [7]. It is a prominent feature of PCOS and affects approximately 80% of its cases [8]. However, the prevalence of IR in women with PCOS is highly inconsistent due to different IR measures and cutoff values [9]. IR is mainly promoted in PCOS via hyperandrogenemia, which alters insulin activity in adipocytes and the skeletal muscle. Furthermore, IR decreases adiponectin concentrations, which also increases the sensitivity of tissues to insulin [10]. Nevertheless, IR is also believed to be one of more important pathogenetic components of PCOS. Insulin, acting on its receptor in the theca cells, stimulates the excessive production of androgens and inhibits the hepatic production of sex hormone binding globulin, which results in an increase in the concentration of free testosterone in the blood. In addition, insulin, by acting on the GnRH (gonadotropin-releasing hormone) receptor in the hypothalamus, increases the effect of LH (luteinizing hormone) on the ovary, which intensifies the excessive production of androgens. These mechanisms indicate that IR and hyperandrogenism are closely related to each other and significantly affect the pathogenesis and course of PCOS [10,11]. 

It is suggested that diet and physical activity significantly influence insulin sensitivity. Importantly, the role of dietary patterns in relation to IR is remarkable and quite well proven [12,13,14,15]. Physical activity, being an integral part of any balanced lifestyle, is recommended for PCOS women. Particularly, vigorous aerobic physical exercise may result in an improvement in insulin sensitivity [16]. Recent recommendations regarding the treatment of PCOS have emphasized the great importance of lifestyle interventions in the management of this disease [17,18]. It is well-known that, in this group of women, reductions of 5% of body weight were associated with the improvement of hormonal and metabolic parameters and a greater chance of spontaneous ovulation [17]. Importantly, lifestyle changes appear to be as effective in treating this disease as metformin. Therefore, it was suggested that lifestyle modifications should be the first line of treatment in PCOS [15]. 

Bearing in mind the fact that IR that accompanies PCOS does not only aggravate the hormonal imbalance and ovulation disorders but it also increases the risk of cardiometabolic complications, it is important to establish the role of a balanced diet and physical activity in the management of IR in this disease. Therefore, in this study we aimed to evaluate the link between diet and physical activity and various indices of insulin resistance, including adipokines secreted by the adipose tissue. 

## 2. Materials and Methods

### 2.1. Participants

This case-control study included 56 women with polycystic ovary syndrome and 33 healthy control women, who were enrolled in the Department of Gynecological Endocrinology of the Medical University of Warsaw in 2021–2022. The inclusion criteria for the study group were as follows: age between 18 and 40 and polycystic ovary syndrome diagnosed according to the Rotterdam diagnostic criteria. They included the presence of at least two of the following three criteria: oligo-/amenorrhea, clinical and/or biochemical hyperandrogenism and polycystic ovaries detected via ultrasound exam [19]. Exclusion criteria were as follows: diabetes; thyroid dysfunction; endometriosis; Cushing’s syndrome; androgen-releasing tumor; congenital adrenal hyperplasia; chronic hypertension, cardiovascular diseases; the use of lipid-lowering, hormonal or insulin-sensitizing drugs; pregnancy; and lactation. The additional exclusion criteria, such as diagnosed epilepsy, an implanted pacemaker or defibrillator and metal endoprostheses, were included due to the method of body composition measurement (BIA—Bioelectrical Impedance Analysis). Women in the control group aged between 18 and 40 were generally healthy with no chronic diseases. They had normal and regular menstrual cycles with no signs of hyperandrogenism and the sonographic appearance of the ovaries was normal. All participating women provided written informed consent to participate in the study. The study was approved by the Ethics Committee of the Medical University of Warsaw (no. KB/170/2019).

### 2.2. Anthropometric Measurements

Body weight and height measurements were performed using standard procedures [20]. Body mass index was calculated as follows: weight (kg)/height2 (m^2^). The interpretation of the ratio was based on the classification specified by the World Health Organization (WHO): <18.5 kg/m^2^—underweight; 18.5–24.9 kg/m^2^—normal weight; 25.0–29.9 kg/m^2^—overweight; and ≥30.0 kg/m^2^—obese [21]. In addition, waist circumference was measured according to WHO recommendations at the midpoint between the lower margin of the least palpable rib and the top of the iliac crest using a stretch-resistant tape [22].

### 2.3. Body Composition Analysis with Bioelectrical Impedance (BIA)

Whole body composition was assessed using the Maltron BioScan 920-II multi-frequency bioelectrical impedance analyzer according to the manufacturer’s instructions (Maltron International Ltd., Rayleigh, UK). According to the European Society of Parenteral and Enteral Nutrition, the women were instructed to follow certain recommendations before undergoing the BIA measurement, i.e., no alcohol and no fluids containing caffeine for 24 h before the test, lack of physical activity for 12 h before the test and empty stomach and bladder before the test [23]. The subjects were measured in the supine position with the limbs separated, 30 degrees away from the body axis, after resting for about 3 min. Before placing the electrodes on the top middle part of the right hand and the top middle part of the right foot, the sites were cleaned using isopropyl alcohol to limit the possible errors and ensure adherence [24]. 

The quantitative analysis of abdominal adipose tissue (subcutaneous and visceral) was performed in a standing position, with the upper limbs away from the body. The configuration of the placement of the electrodes was strictly defined by the manufacturer of the device [24]. Based on the waist circumference and the impedance of the visceral and abdominal tissue, the following parameters were determined: subcutaneous fat surface (SAT in cm^2^), visceral fat surface (VAT in cm^2^) and visceral-to-subcutaneous fat ratio (VAT/SAT ratio).

### 2.4. Biochemical Analysis

After 12-h fasting, venous blood was collected from each participant during the follicular phase (2–6 days) of the menstrual cycle between 7 am and 9 am. To obtain the serum, the samples were centrifuged at 2500× *g* for 10 min at 4 °C. On the day of blood collection, serum glucose and insulin levels were determined. The rest of the serum was divided into small portions and stored for no longer than 3 months at −80 °C until the remaining biochemical tests were performed.

Serum glucose level was assayed via the enzymatic method with hexokinase (Integra 400 plus analyzer, Roche Diagnostics, Basel, Switzerland), whereas serum insulin concentrations were analyzed using two-step chemiluminescent microparticle immunoassay (CMIA; Alinity I analyzer; Abbott Diagnostics GmbH, Wiesbaden, Germany).

To assess insulin resistance, the following mathematical models were used: HOMA-IR (the homeostatic model assessment of insulin resistance calculated as: [fasting insulin (µU/mL) × fasting glucose (mg/dL)]/405); HOMA-AD (the homeostatic model assessment-adiponectin calculated as: [fasting plasma insulin (μU/mL) × fasting glucose (mmol/L)/adiponectin (μg/mL)] and L/A (leptin-to-adiponectin ratio calculated as: leptin (ng/mL)/adiponectin μg/mL). The defined cutoff points for different insulin resistance indices used were HOMA-IR ≥ 2.5 [25], HOMA-AD ≥ 6.26 [26] and L/A > 2.2 [27]. 

The concentrations of serum adipokines were determined via commercial enzyme-linked immunosorbent assay (ELISA) according to the manufacturer`s instructions. Leptin was assessed using a kit from DRG Instruments GmbH (Marburg, Germany). The analytical sensitivity of the assay was 0.7 ng/mL, while intra- and inter-assay coefficients of variation (CV) were 4.2–7.3% and 3.7–9.1%*,* respectively. Total adiponectin was determined using a kit manufactured by TECOmedical AG (Sissach, Switzerland) with a lower detection limit below 0.27 ng/mL, intra-assay CV of 3.14–3.67% and inter-assay CV of 6.93–8.16%. Resistin was measured using a kit manufactured by Mediagnost (Reutlingen, Germany); the analytical sensitivity of the assay was 0.012 ng/mL, while the intra- and inter-assay CV were 4.49–4.97% and 3.37–6.67%, respectively.

### 2.5. Nutritional Assessment 

The assessment of the women’s nutrition was based on the original food frequency questionnaire (FFQ), which was conducted during a face-to-face interview with a qualified nutritionist. The FFQ consisted of 15 items which have a negative or positive relationship with the sensitivity of tissues to insulin. The main food products included wholegrain cereal products, refined cereal products, natural yoghurt, fruits, vegetables, vegetable oils (including avocado and soft margarines), animal fats (including butter, solid margarines, palm oil and coconut oil), nuts, legumes, red meat, processed red meat, fatty sea fish, sweet drinks, sweets (including sugar) and fast food. The participants were asked to report their average frequency (none, daily, weekly or monthly) and portion sizes for consumption of each food item. The sizes of declared food portions were verified using the “Album of Photographs of Food Products and Dishes” from the National Food and Nutrition Institute [28].

Based on the awarded points (0, 0.5 or 1), the score of the adherence to the diet recommended in insulin resistance was calculated. A greater number of points received by a subject translated into greater compliance with the diet, lowering insulin resistance. The maximum number of points that could be obtained was 15.

### 2.6. Physical Activity 

Physical activity was measured using an Actigraph GT3X-BT activity monitor (Actigraph Corp, Pensacola, FL, USA) worn for 7 consecutive days at an elastic belt around the waist and positioned on the right hip. Raw accelerometer data were downloaded using ActiLife software (version 6.13.0, ActiGraph Corporation). The intensity of physical activity was measured using cut points for adults established by Freedson et al. [29]. To estimate the level of physical activity, the numbers of total minutes of moderate, vigorous and moderate-to-vigorous physical activity (MVPA) per week were recorded.

### 2.7. Statistical Analysis

All calculations were performed with STATISTICA TM 13.3 software (TIBCO Software, Palo Alto, CA, USA). Variable distributions were evaluated with the D’Agostino’s K-squared test and descriptive statistics (means, standard deviations, medians and interquartile ranges) were calculated. Due to the fact that the Study and Control Groups were not of equal sizes, the Mann–Whitney–Wilcoxon test was used to compare differences between them. The contingency tables with the Fisher’s exact test or chi-square test were used to assess the relationship between the frequency of insulin resistance in various measures. 

Linear regression analysis was used to assess the relationship between the examined parameters and physical activity or diet scores. The least square estimation was used to estimate parameters for linear regression. A standardized regression coefficient (β) with a 95% confidence interval (95% CI) was estimated for each independent variable included in the model. Logistic regression analysis was performed in order to examine the chances of normal values of the examined parameters depending on the scores for diet or physical activity. The maximum likelihood method of estimation was used to estimate parameters for logistic regression. For each independent variable included in the model, an odds ratio (OR) with a 95% confidence interval (95% CI) was estimated. Correlations between variables were examined using Pearson’s correlation coefficient. *p*-values of <0.05 were considered statistically significant. 

## 3. Results

### 3.1. Characteristics of PCOS and Healthy Women

The characteristics of PCOS and control women, i.e., the age and height, were comparable and were not significantly different. As expected, the anthropometric and body composition data showed several differences between the groups. Women with PCOS had significantly higher body weight (*p* = 0.008), BMI (*p* = 0.004), waist circumference (*p* = 0.003), total fat mass (*p* = 0.008), visceral fat (*p* = 0.018) and subcutaneous fat (*p* = 0.003) compared to healthy control women. Interestingly, the VAT/SAT ratio was also higher in this group, though the difference was not statistically significant.

Biochemical blood parameters also showed differences between groups. PCOS women had a significantly lower concentration of adiponectin compared to control women (7.57 ± 2.44 vs. 14.15 ± 3.98 μg/mL; *p* < 0.001). However, we did not observe any significant differences in leptin and resistin levels. Women with PCOS had significantly higher L/A ratios compared to healthy women (*p* < 0.001). Interestingly, women from the Study Group had lower HOMA-IR values compared to the Control Group, though the difference was not statistically significant. This result was in contrast to another measure of insulin resistance, i.e., HOMA-AD, where the results clearly differed between the groups. PCOS women were characterized by significantly higher HOMA-AD compared to control women (4.11 ± 3.86 vs. 3.29 ± 1.37; *p* = 0.029). 

PCOS Group was characterized by significantly lower MVPA compared to the Control Group (301.92 ± 107.67 vs. 376.60 ± 43.72 min/week; *p* = 0.003). Similarly, moderate and vigorous physical activity levels were also significantly lower (*p* = 0.012; *p* = 0.016, respectively). We did not observe significant differences between the diet scores between the Study and Control Groups (*p* = 0.280). The detailed comparisons of anthropometric data, body composition measures, biochemical parameters and the diet score are shown in Table 1. 

### 3.2. Insulin Resistance and Correlation between HOMA-IR and HOMA-AD

The Pearson’s correlation coefficient revealed a strong positive correlation between HOMA-IR and HOMA-AD in the Study and Control Groups (*r* = 0.8248, *p* < 0.001; *r* = 0.6662, *p* = 0.00002, respectively). Moreover, the correlation was much stronger in the group of women with PCOS. 

According to the HOMA-IR index, 11 women with PCOS and 10 women from the Control Group were characterized by abnormal tissue sensitivity to insulin. Regarding the HOMA-AD index, as many as 22 women with PCOS had insulin resistance, while only 3 women had a score ≥ 6.26 in the Control Group. In addition, we noted that the probability of an abnormal HOMA-AD result was significantly increased in PCOS Group (OR 6.47, 95% CI 1.76–23.80; *p* = 0.0029). Finally, the L/A ratio used to assess insulin resistance showed that 25 women with PCOS and only two women from the Control Group had abnormal results. 

In addition, we observed a relationship between the normal result obtained in HOMA-IR and HOMA-AD in both PCOS (*χ*^2^ = 10.76; *p* = 0.00104) and Control Groups (*χ^2^* = 15.04; *p* = 0.00011). As regards women with PCOS, 11 had insulin resistance according to both indices, with only two such patients being found in the Control Group.

### 3.3. Effect of Diet and Physical Activity on Adipokines and Insulin Resistance

Linear regression analysis showed a differential effect of physical activity on the HOMA-IR value depending on the group. We observed that higher levels of physical activity (expressed as MVPA) were associated with lower HOMA-IR in PCOS Group (*t* = −2.109; *p* = 0.038). However, there was no statistically significant relationship between those parameters in the Control Group. Moreover, there was no relationship between HOMA-IR and diet score both in PCOS and Control Groups. Additionally, no such relationship was observed between physical activity or the diet score and adipokines, HOMA-AD and the L/A ratio in both groups of women. Adjustment for BMI and MVPA did not affect the results. 

In addition, logistic regression showed that there was a differential effect of physical activity on the normal level of HOMA-IR depending on the group. In the PCOS Group, higher levels of physical activity (expressed as MVPA) translated into a greater chance of a normal HOMA-IR level (OR 1.012, 95% CI 1.003–1.021; *p* = 0.010). However, no such relationship was observed in the Control Group. In addition, the diet score did not have an effect on normal HOMA-IR value in PCOS and control women. Similarly, no such link was observed between physical activity or the diet score and adipokines, HOMA-AD and the L/A ratio in both groups of women. Similarly to the previous analysis, adjustment for BMI and MVPA did not change the outcomes.

### 3.4. Correlations between Adipokines, Insulin Resistance Markers and Anthropometric Measurements and Body Composition

The analysis of the group of PCOS women revealed negative-to-moderate correlations between serum adiponectin and weight (*r* = −0.5997; *p* < 0.001), BMI (*r* = −0.6057; *p* < 0.001), waist circumference (*r* = −0.6034; *p* < 0.001), fat mass (*r* = −0.5835; *p* < 0.001), VAT (*r* = −0.6508; *p* < 0.001), SAT (*r* = −0.5232; *p* < 0.001) and VAT/SAT ratio (*r* = −0.5187; *p* < 0.001). In this group, serum leptin and resistin were also significantly related to all the anthropometric and body composition measurements (moderate-to-strong positive correlations). As regards non-PCOS women, we noted that only leptin was significantly associated with anthropometric and body composition measurements and the correlations were mostly stronger than in PCOS Group.

Fasting insulin and fasting glucose of PCOS women correlated positively with all measurements, except for fat free mass where the correlation was inverse (*r* = −0.6014, *p* > 0.001 and *r* = −0.5437, *p* < 0.001, respectively). As for the Control Group, only the correlation between fasting insulin and SAT was statistically significant (*r* = 0.5100, *p* = 0.02). In case of fasting glucose, none of the relationships were significant.

All IR indices were correlated with anthropometric and body composition measurements in PCOS women. HOMA-IR and the A/L ratio were the most strongly correlated with VAT (*r* = 0.6061, *p* < 0.001 and *r* = 0.7305, *p* < 0.001, respectively). Interestingly, HOMA-IR was only correlated with SAT (*r* = 0.5239, *p* = 0.002) in the Control Group. In PCOS Group, we noted the strongest positive correlation between HOMA-AD and weight (*r* = 0.7197, *p* < 0.001), BMI (*r* = 0.7012, *p* < 0.001) and VAT (*r* = 0.7185, *p* < 0.001). Other HOMA-AD correlations were of moderate strength. In the Control Group, the correlations of HOMA-AD with anthropometric and body composition parameters were generally much weaker. Additionally, in PCOS women HOMA-AD was correlated with VAT/SAT ratio (*r* = 0.6307, *p* < 0.001), whereas no such link was observed in non-PCOS women. We noted a similar relationship in case of the A/L ratio. Correlations between adipokines, insulin resistance markers and anthropometric measurements and body composition in PCOS and non-PCOS women are presented in detail in Table 2.

## 4. Discussion

It is well-proven that PCOS is strongly associated with carbohydrate metabolism disorders, especially with IR. It was observed that this group of patients was up to seven times more likely to develop type 2 diabetes mellitus and impaired glucose tolerance compared to healthy women [30]. According to numerous studies, PCOS women had higher fasting insulin and HOMA-IR compared to healthy non-PCOS women [7,31,32,33,34,35,36]. Contrary to most studies, our publication and studies by Mishra et al. [37], Seow et al. [38] and Pandit et al. [39] did not show any significant differences between those parameters in the Study and Control Groups. Notably, due to the fact that insulin and HOMA-IR are closely correlated with androgens some authors suggested that IR was only particularly intense in PCOS patients with severe hyperandrogenism [10,40]. Moghetti et al. [40] reported a higher prevalence of IR (measured by hyperinsulinemic-euglycemic glucose clamp) observed in the PCOS phenotype associated with hyperandrogenism. Barrea et al. [41] also emphasized that the exacerbation of IR measured by HOMA-IR was different due to the metabolic status of the examined PCOS women. Metabolically healthy obese PCOS women had significantly lower fasting insulin and HOMA-IR compared to metabolically unhealthy obese PCOS women. Additionally, it should be noted that HOMA-IR is not the most accurate method for assessing IR [9]. The above mentioned factors may explain why different results were obtained in our study and show that other factors influencing IR in women with PCOS should be considered, with particular emphasis on androgenization and abdominal obesity. 

The early diagnosis and management of IR in women with PCOS is one of the most important aspects of treating this disease. However, there are no recommendations regarding the assessment of IR in this group of women. The hyper-insulinemic–euglycemic glucose clamp is the gold standard method for assessing insulin sensitivity. Nevertheless, due to the fact that it is very complex, invasive and impractical, the method is mainly used for research purposes [8]. Amisi et al. [8] reviewed surrogate IR assessment methods in PCOS, which would be much easier to use in clinical practice. One of the methods mentioned in this review was linked to adipokines, which are involved in the regulation of tissue insulin sensitivity and HOMA-IR. 

In the present study, apart from HOMA-IR we used HOMA-AD to evaluate IR. To our knowledge, our study is the first one that uses HOMA-AD in a group of women with PCOS. HOMA-AD is a very promising marker for IR assessment because it also includes adiponectin, which is involved in the regulation of insulin sensitivity. This surrogate IR marker was first validated in Japanese adults and presented a stronger correlation with the euglycemic–hyper-insulinemic clamp technique than HOMA-IR (*r* = −0.643, *p* < 0.001; *r* = −0.591, *p* < 0.001, respectively) [42]. A study by Vilela et al. [43] conducted in adult women with metabolic syndrome also revealed a stronger correlation between HOMA-AD and euglycemic–hyper-insulinemic clamp compared to HOMA-IR, which suggests that it may be a more sensitive IR marker. Our study demonstrated a strong positive correlation between HOMA-IR and HOMA-AD in the Study and Control Groups. Moreover, the correlation was much stronger in the group of women with PCOS. In contrast to HOMA-IR, we observed significantly higher HOMA-AD values in the PCOS Group compared to non-PCOS Group. Additionally, when we used HOMA-AD we observed a higher prevalence of IR among PCOS women.

In our research, we also proposed the L/A ratio to assess IR in PCOS women. It was previously suggested by Larsen et al. [27] that, in obese adults, the L/A ratio could be a potential biomarker for IR. As regards PCOS women, it was confirmed that the L/A ratio correlated positively with HOMA-IR [44,45,46]. Furthermore, Golbahar et al. [47] demonstrated that the L/A ratio was independently associated with PCOS, indicating its high sensitivity and specificity for the diagnosis of PCOS. We observed that the L/A ratio was significantly higher in PCOS women compared to their healthy counterparts. Several other authors obtained similar results [45,47,48,49]. Moreover, Savastano et al. [48] reported that the L/A ratio was significantly higher in lean and obese PCOS women compared to lean and obese controls, which indicates the possibility of using this indicator to assess IR independently of BMI. However, it would be worth considering the influence of abdominal obesity on this parameter.

The importance of physical activity in the prevention and treatment of many chronic diseases is well documented [50]. Studies in women with PCOS showed that physical activity had a beneficial effect on the hormonal profile, ovulation and regulation of the menstrual cycle, which translated directly into reproductive health and pregnancy rates [51,52]. Our study also showed that physical activity should be an integral part of PCOS treatment due to its beneficial effect on insulin resistance. We reported that higher physical activity was related to lower HOMA-IR in the PCOS Group. Moreover, higher levels of physical activity were associated with greater chances of attaining normal HOMA-IR values. A systematic review of 46 studies revealed that vigorous aerobic exercise improved insulin sensitivity in women with PCOS [16]. This finding was also confirmed by two recent systematic reviews and meta-analyses [53,54]. Turan et al. [55] found a significant decrease in HOMA-IR in non-overweight PCOS women after 8 weeks of structured exercise, while in the Control Group no difference was observed. Several studies using HOMA-IR revealed a correlation between exercise and insulin sensitivity, where the authors found a significant decrease in insulin resistance in the PCOS Group [56,57,58,59]. Some researchers also observed an improvement in insulin sensitivity but measured with different methods than HOMA-IR [31,60,61]. Changes were also observed independently of weight loss. 

No relationship was observed between the amount of physical activity and the concentrations of all tested adipokines (adiponectin, leptin and resistin). The results obtained by Stener-Victorin et al. [62], Covington et al. [60] and Almenning et al. [57], where the concentration of adiponectin was unrelated to physical activity, were similar to our findings. Contrary to our results, Al Eisa et al. [59] noted that 12 weeks of moderate aerobic training had a significant positive effect on adiponectin levels in PCOS women. Another study in PCOS women showed that 12 weeks of HITT exercise contributed to an increase in adiponectin concentration, while the level of leptin remained unchanged [63]. Souza et al. [58] found that 8 weeks of aerobic activity resulted in an increase in adiponectin and a decrease in leptin, albeit only in lean women with PCOS. Covington et al. [60] observed that 8 weeks of aerobic exercise caused a decrease in the ratio of leptin to high-molecular-weight adiponectin, while leptin to total adiponectin ratio was unchanged. It is believed that the effects of exercise on adipokine levels may occur more quickly in non-overweight than overweight PCOS women and reduction in body weight is the most important factor influencing adipokine concentration. Furthermore, changes in the visceral adipose tissue as a result of physical activity are the most important factors reinforcing these relationships [58,63]. These mechanisms may explain the lack of the influence of physical activity on adipokines in our study, as well as HOMA-AD and the L/A ratio. Therefore, in the future interventional study should be carried out to determine whether different intensities of physical activity are associated with changes in adipokines and tissue insulin sensitivity.

The importance of diet, in addition to physical activity, was clearly emphasized in the recommendations regarding the treatment of PCOS [17,18]. Despite this fact, the eating habits of women with PCOS show many abnormalities. Several papers revealed that the diet and lifestyle of PCOS patients was poor compared to the control group [36,64,65]. However, our study revealed no statistically significant difference between dietary patterns in PCOS and non-PCOS women. A systematic review and meta-analysis of 19 studies highlighted that a balanced diet in PCOS contributed to better insulin sensitivity (measured via HOMA-IR) [15]. The Mediterranean diet, DASH diet (Dietary Approaches to Stop Hypertension) and low glycemic index diet seem to be the healthiest dietary approaches that have the strongest relationship with greater insulin sensitivity among PCOS women [12,13,14,15]. A study by Bykowska-Derda et al. [3] showed that PCOS women with low adherence to the western dietary pattern were at a lower risk of having IR. In particular, high intake of meat was associated with metabolic disorders in PCOS. Interestingly, dietary patterns rich in plant products and physical activity were related to improvement in metabolic health among PCOS women. According to a study by Nybacka et al. [66], 4 months of dietary intervention (well-balanced diet with caloric restrictions) was associated with an improvement in HOMA-IR. In our study, we proposed assessing the diet using a diet score, which comprised the effects of dietary factors and a negative and positive impact on insulin sensitivity. However, higher adherence to the diet that increased insulin sensitivity (measured by diet score) was not found to be associated with HOMA-IR, HOMA-AD, A/L ratio and adipokine levels, even though adiponectin concentration was also influenced through dietary patterns and particular products and nutrients in the diet [5]. Our results are similar to those obtained by several other authors [1,67,68]. Wang et al. [1] reported that HOMA-IR values did not differ in PCOS women after 6 months of intervention (balanced diet with caloric restriction and moderate physical activity three times per week). Shishehgar et al. [68] observed that hypocaloric low glycemic index diet was unrelated to an improvement in insulin sensitivity (measured via HOMA-IR). Nybacka et al. [67] also did not note a change in HOMA-IR values as a result of a well-balanced diet developed individually by a dietitian. Dâmaso et al. [69] observed no influence of dietary modifications on adiponectin concentration and HOMA-AD values in overweight and obese women. However, they observed a significant improvement in leptin concentrations. When analyzing research results, it should be emphasized that lifestyle modification alone seems to be insufficient; satisfactory results are achieved only after combining diet and physical activity with pharmacological treatment in some patients with PCOS. Moreover, available evidence suggests that the adiponectin-mediated relationship between exercise, diet and insulin sensitivity is more likely at the level of receptor expression, which may explain why lifestyle modifications do not immediately translate into laboratory results [70]. Due to the unclear results of research concerning the relationship between diet and the course of PCOS, it seems reasonable to conduct an intervention study assessing the impact of this parameter on the metabolic profile of women with PCOS, with particular emphasis on IR. 

The use of an accelerometer for the precise measurement of physical activity was a strength of our study. However, this study had several limitations. Firstly, the size of the Study and Control Groups was small. Secondly, the use of the original food frequency questionnaire based on declared frequency and portion sizes by the participants might lead to the under- or over-estimation of dietary intake. Finally, our population was mainly Caucasian, with the participants holding a university education and high socioeconomic status, which could significantly reduce the representativity of the study. 

## 5. Conclusions

In conclusion, our study supports the role of physical activity in the management of insulin resistance in PCOS women. Due to the fact that physical activity promotes very important metabolic changes, all women with PCOS should be given the general advice to be physically active. However, due to the small size of the Study and Control Groups the role of diet in the treatment of insulin resistance in PCOS cannot be unequivocally stated; the same is true of the role of lifestyle intervention in modulating adiponectin, leptin and resistin concentrations. Moreover, our findings indicated that HOMA-AD might be a promising surrogate marker for insulin resistance assessment in women with PCOS. Therefore, studies in a larger group of PCOS patients are particularly needed to establish the role of HOMA-AD in the diagnosis of insulin resistance and the role of diet and physical activity in treating this metabolic disorder. 

## Figures and Tables

**Table 1:** Comparison of anthropometric, body composition, biochemical parameters, physical activity and diet score between PCOS Group and Control Group.

Parameters	PCOS (n = 56)	CONTROL (n = 33)	*p*-Value *
Age and Anthropometric Measurements			
Age (years)	25.96 ± 4.10	29.12 ± 6.85	NS
	25.00 (19–38)	31.00 (19–38)	
Weight (kg)	75.99 ± 21.46	63.09 ± 10.44	0.008
	70.00 (45.0–126.4)	60.00 (47.0–93.0)	
Height (cm)	166.70 ± 5.12	167.42 ± 5.85	NS
	165.00 (158–180)	167.00 (154–178)	
BMI (kg/m^2^)	27.25 ± 7.40	22.43 ± 3.17	0.004
	24.65 (17.2–42.9)	21.90 (17.9–32.9)	
WC (cm)	86.84 ± 18.34	75.20 ± 8.12	0.003
	84.50 (58–126)	73.50 (63–91)	
Body Composition			
FM (kg)	27.93 ± 15.81	18.34 ± 7.44	0.008
	23.24 (7.45–64.37)	16.75 (8.94–40.55)	
FM (%)	34.08 ± 10.62	28.04 ± 6.66	0.010
	33.16 (15.84–51.96)	27.92 (16.25–43.60)	
VAT (cm^2^)	154.32 ± 124.21	72.52 ± 42.24	0.018
	112.50 (21–350)	57.00 (22–174)	
SAT (cm^2^)	149.68 ± 87.86	94.39 ± 39.99	0.003
	126.00 (37–380)	88.00 (28–212)	
VAT/SAT	1.03 ± 0.58	0.77 ± 0.30	NS
	0.87 (0.38–2.41)	0.74 (0.33–1.45)	
FFM (kg)	48.06 ± 6.14	44.91 ± 3.93	0.021
	46.76 (37.40–64.07)	44.77 (37.25–53.79)	
FFM (%)	65.99 ± 10.51	71.96 ± 6.66	0.010
	66.84 (48.05–84.16)	72.08 (56.40–83.75)	
MM (kg)	21.07 ± 2.92	19.51 ± 1.76	0.012
	20.46 (14.76–28.34)	19.52 (16.22–23.71)	
BCM (kg)	25.24 ± 4.41	22.87 ± 2.35	0.015
	24.2 (15.96–35.13)	22.9 (18.79–28.36)	
ECM (kg)	22.78 ± 2.10	22.05 ± 1.92	NS
	22.7 (19.00–28.94)	21.8 (18.45–25.92)	
TBW (%)	46.50 ± 5.20	49.75 ± 3.99	0.004
	46.37 (37.50–55.88)	49.65 (41.3–57.61)	
ECW (%)	47.15 ± 3.74	48.29 ± 3.36	0.002
	46.70 (44.32–67.97)	47.53 (45.63–61.95)	
ICW (%)	52.86 ± 3.75	51.70 ± 3.36	0.002
	53.30 (32.02–56.67)	52.46 (38.04–54.36)	
ECW/ICW	0.91 ± 0.20	0.94 ± 0.16	0.002
	0.88 (0.80–2.12)	0.91 (0.84–1.63)	
Biochemical Parameters			
Fasting glucose (mg/dL)	91.63 ± 6.98	96.45 ± 6.26	<0.001
	90.50 (77.4–122.0)	96.00 (82.0–113.0)	
Fasting insulin (μU/mL)	7.71 ± 4.39	8.24 ± 3.73	NS
	6.50 (2.50–24.07)	7.50 (3.40–18.00)	
Adiponectin (μg/mL)	8.13 ± 3.51	14.70 ± 5.92	<0.001
	7.57 (2.20–16.40)	14.15 (5.02–27.50)	
Leptin (ng/mL)	14.42 ± (10.77)	10.46 ± 9.25	NS
	10.31 (1.40–48.20)	6.60 (2.01–43.58)	
Resistin (ng/mL)	7.29 ± 2.50	7.41 ± 1.75	NS
	6.72 (4.10–15.70)	6.93 (4.73–12.14)	
HOMA-IR	1.77 ± 1.08	1.97 ± 0.92	NS
	1.38 (0.54–5.59)	1.70 (0.80–4.43)	
HOMA-AD	6.70 ± 5.97	3.58 ± 2.35	0.029
	4.11 (1.17–23.98)	3.29 (0.99–11.63)	
L/A	2.60 ± 2.74	0.93 ± 1.25	<0.001
	1.40 (0.12–12.06)	0.56 (0.13–6.55)	
Diet Score and Physical Activity			
Diet score	7.56 ± 2.46	8.15 ± 2.10	NS
	7.50 (1.5–13.0)	8.50 (3.5–12.0)	
Moderate [min/week]	296.06 ± 137.94	372.41 ± 139.28	0.012
	264.33 (71.50–611)	359.10 (158.17–821.50)	
Vigorous [min/week]	20.24 ± 36.31	35.34 ± 45.72	0.009
	8.50 (0.00–223.67)	16.80 (2.80–203.83)	
MVPA [min/week]	316.30 ± 141.42	407.67 ± 135.78	0.003
	301.92 (72.17–627.33)	376.60 (186.80–835.33)	

Data are mean ± standard deviation, median and interquartile ranges. * Mann–Whitney test; *p* < 0.05. Abbreviations: NS—not statistically significant; BMI—body mass index; WC—waist circumference; FM—fat mass; VAT—visceral fat surface; SAT—subcutaneous fat surface; VAT/SAT—visceral to subcutaneous fat ratio; FFM—fat free mass; MM—muscle mass; BCM—body cell mass; ECM—extracellular matrix; TBW—total body water; ECW—extracellular water; ICW—intercellular water, ECW/ICW—extracellular water-to-intercellular water ratio; HOMA-IR—homeostatic model assessment of insulin resistance; HOMA-AD—homeostatic model assessment—adiponectin; L/A—leptin-to-adiponectin ratio; and MVPA—moderate-to-vigorous physical activity.

**Table 2:** Correlation between adipokines, insulin resistance markers and anthropometric measurements and body composition.

Parameters	Adiponectin		Leptin		Resistin		Fasting Insulin		Fasting Glucose		HOMA-IR		HOMA-AD		A/L	
	PCOS	Control	PCOS	Control	PCOS	Control	PCOS	Control	PCOS	Control	PCOS	Control	PCOS	Control	PCOS	Control
Weight (kg)	−0.5997 *	−0.2335	0.6896 *	0.7141 *	0.6133 *	0.0802	0.6041 *	0.2121	0.5819 *	0.0998	0.5859 *	0.2177	0.7197 *	0.5340 *	0.6807 *	0.7304 *
BMI (kg/m^2^)	−0.6057 *	−0.1443	0.7208 *	0.7676 *	0.6034 *	0.1274	0.6062 *	0.2520	0.5419 *	0.0784	0.5903 *	0.2516	0.7012 *	0.5169 *	0.6979 *	0.7393 *
WC (cm)	−0.6034 *	−0.2544	0.7154 *	0.6545 *	0.5639 *	0.0706	0.6147 *	0.1175	0.5715 *	0.1690	0.5984 *	0.1404	0.6983 *	0.4317 *	0.6907 *	0.5929 *
FM (%)	−0.5835 *	0.0914	0.7437 *	0.7838 *	0.5843 *	0.1219	0.6139 *	0.3266	0.5412 *	0.1059	0.5823 *	0.3240	0.6735 *	0.4727 *	0.6958 *	0.6481 *
VAT (cm^2^)	−0.6508 *	0.1500	0.7396 *	0.7544 *	0.4915 *	0.0486	0.6224 *	0.2457	0.5575 *	0.1170	0.6061 *	0.2572	0.7185 *	0.4746 *	0.7305 *	0.6371 *
SAT (cm^2^)	−0.5232 *	0.2665	0.6736 *	0.8015 *	0.4339 *	0.1994	0.6038 *	0.5100 *	0.4743 *	0.1871	0.5742 *	0.5239 *	0.5766 *	0.6985 *	0.5949 *	0.7710 *
VAT/SAT	−0.5187 *	0.1072	0.5978 *	0.1897	0.4688 *	−0.1556	0.5616 *	−0.2847	0.5042 *	0.0313	0.5401 *	−0.2794	0.6307 *	−0.1145	0.5890 *	0.0860
FFM (%)	0.5852 *	0.0910	−0.7451 *	−0.7844 *	−0.5933 *	−0.1212	−0.6014 *	−0.3268	−0.5437 *	−0.1060	−0.5886 *	−0.3242	−0.6805 *	−0.4729 *	−0.6992 *	−0.6486 *
MM (kg)	−0.5526 *	−0.2201	0.5457 *	0.4099 *	0.5778 *	0.0493	0.5265 *	0.0539	0.5236 *	0.0438	0.5170 *	0.0603	0.6707 *	0.4017 *	0.6598 *	0.5530 *

Data are presented as Pearson’s r coefficients; * *p* < 0.05. Abbreviations: HOMA-IR—homeostatic model assessment of insulin resistance; HOMA-AD—homeostatic model assessment—adiponectin; L/A—leptin-to-adiponectin ratio; BMI—body mass index; WC—waist circumference; FM—fat mass; VAT—visceral fat surface; SAT—subcutaneous fat surface; VAT/SAT—visceral-to-subcutaneous fat ratio; FFM—fat free mass; and MM—muscle mass.

## Data Availability

The data analyzed during the current study are available from the corresponding author on reasonable request.
